# The complete mitochondrial genome of *Cyphocaris challengeri* (Amphipoda: Cyphocarididae)

**DOI:** 10.1080/23802359.2023.2270206

**Published:** 2023-10-25

**Authors:** Chloe N. Rabinowitz, Shannon D. Brown, Sean M. McAllister, Amanda K. Winans, Julie E. Keister, Matthew P. Galaska

**Affiliations:** a School of Environmental and Forest Sciences, University of Washington, Seattle, WA, USA; b Cooperative Institute for Climate, Ocean, & Ecosystem Studies, University of Washington, Seattle, WA, USA; c Pacific Marine Environmental Laboratory, National Oceanographic and Atmospheric Administration, Seattle, WA, USA; d School of Oceanography, University of Washington, Seattle, WA, USA

**Keywords:** Mitogenome, mtDNA, next-generation sequencing, Lysianassoidea

## Abstract

The amphipod *Cyphocaris challengeri* is a globally distributed, highly abundant species of zooplankton. Here, we report the complete mitochondrial genome of *C. challengeri* obtained using the Illumina sequencing platform from a specimen collected from Puget Sound, Washington. The mitogenome is a circular DNA molecule with a size of 14,338 bp and 26.7% GC content, with 13 protein-encoding genes, 2 rRNAs, and 22 tRNAs annotated. A maximum likelihood phylogenetic analysis including *C. challengeri* and all other available mitogenomes from Amphipoda places our mitogenome firmly within the Lysianassoidea superfamily, as expected. The newly described mitochondrial genome of *C. challengeri* fills a gap in valuable reference data for detecting this organism using molecular methods such as environmental DNA.

## Introduction

The genus *Cyphocaris* Boeck 1871 includes pelagic and demersal amphipods with a cosmopolitan distribution (Lowry and Stoddart 2003). Twenty species are recognized within this genus (WoRMS Editorial Board 2022), including *Cyphocaris challengeri* Stebbing 1888, which is distributed worldwide (Bowman and McCain 1967). *Cyphocaris challengeri* is the most abundant gammarid amphipod in the mesopelagic zone of the subarctic Pacific, and the species plays an important role in the pelagic food web (Bowman and McCain 1967; Yoo Il, 1970). This carnivorous amphipod species consumes zooplankton, therefore serving as a trophic link between smaller organisms and larger zooplankton predators (Haro-Garay 2003). Additionally, *C. challengeri* have a high essential fatty acid content, which indicates they are a nutrient-rich food source for their predators (Costalago et al. 2020; Hiltunen et al. 2022). Currently, there are no mitochondrial genomes available for the Family Cyphocarididae Lowry & Stoddart 2003, and no available sequences for *C. challengeri.* In this study, we detail the complete mitogenome of *C. challengeri* to provide insight into the evolutionary history and phylogenetic relationships.

## Materials and methods

**Figure 1.**
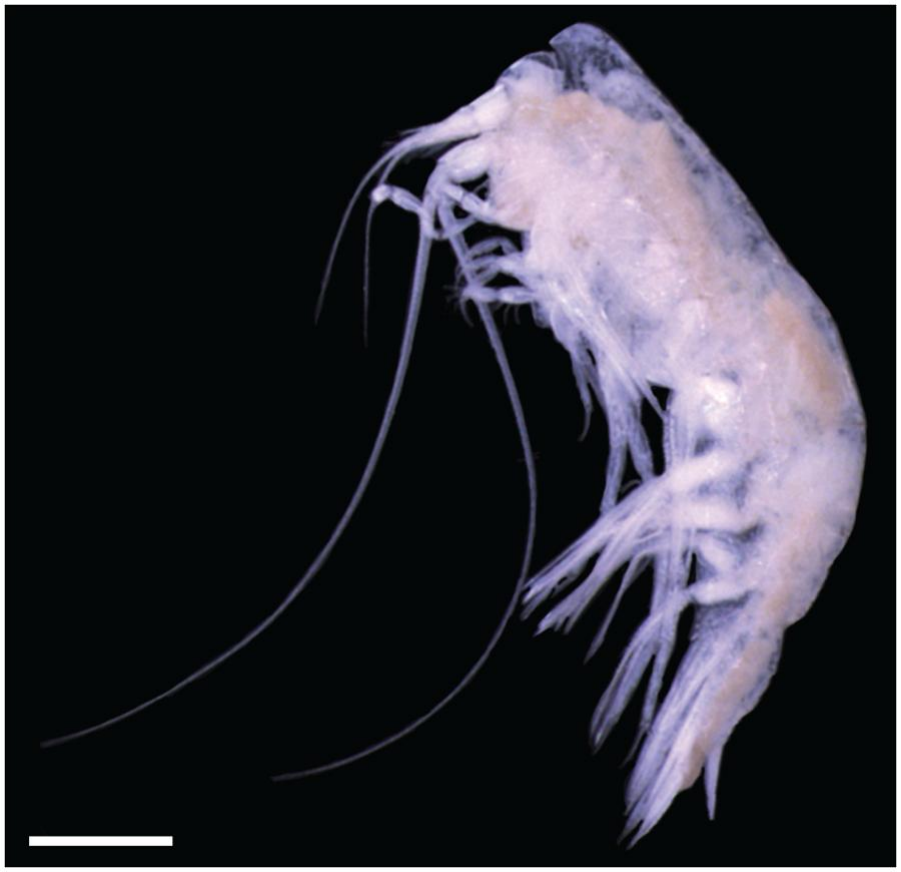
Photograph of the *Cyphocaris challengeri* specimen sequenced in this study. Scale bar 1 mm. Photo taken by C. Rabinowitz.

Eight *C. challengeri* individuals were collected from Carr Inlet, Puget Sound (47°16’35.76” N, 122°42’29.52” W) on 15 September 2019 at 17:18 UTC using a 335-μm mesh bongo net towed obliquely to 90 m depth at the 105 m deep station P38. Specimens were identified by A. Winans, a trained taxonomist in J. Keister’s laboratory at the University of Washington, comparing characteristics with Hughes and Lowry (2015) taxonomic key (whole organism photographed for Figure 1). Genomic DNA was extracted from the body tissue of one whole specimen (T367) using the DNeasy Blood and Tissue kit (Qiagen, Hilden, Germany), according to the manufacturer’s instructions. DNA quality was checked on a 1.5% agarose gel and quantified using a Qubit 4 Fluorometer. The DNA extract is archived with the Ocean Molecular Ecology group at the NOAA Pacific Marine Environmental Laboratory (contact corresponding author) under specimen identification T367. High-throughput paired-end 2 × 150 bp sequencing was performed at Azenta Life Sciences (South Plainfield, NJ) on an Illumina HiSeq 4000 after library prep with the NEBNext Ultra DNA Library Prep Kit, producing 48.9 million raw paired reads totaling 14.7 Gbp.

Raw reads were trimmed to remove adaptors using Trimmomatic 0.39 (Bolger et al. 2014) and then merged using Flash 1.2.11 (Magoc and Salzburg, 2011). Adaptor-trimmed merged and remaining unmerged reads were then quality-filtered using a 4 bp Q20 sliding window in Trimmomatic. Quality filtering produced 31.4 million paired reads and 16.7 million unpaired reads (including both merged and quality filtered unpaired reads) totaling 12.6 Gbp. Quality-controlled reads were then assembled using SPAdes 3.15.5 with k-mer sizes at 21, 55, 85, 115, and 127 bp assuming an isolate genome (Prjibelski et al. 2020). A complete, circular mitogenome with 12.7x coverage was pulled from this assembly using GetOrganelle (Jin et al. 2020). Accuracy of the completed mitogenome was confirmed by aligning the quality-controlled reads (Q30 mapping score) to the final mitogenome product using Bowtie 2 (Supplemental Figure 1) (Langmead and Salzberg 2012). Mitogenome annotation was completed using the MITOS web server (Bernt et al. 2013), with additional annotation using MitoZ (Meng et al. 2019) to identify the *trnL1* gene. Small and large subunit rRNA genes were annotated by hand using reference amphipod mitogenomes aligned to rRNA secondary structures (Pons et al. 2014). Visualizations of the mitogenome annotation were modified from outputs generated by Geneious Prime (v.2022), including standard percent GC calculations.

To confirm the phylogenetic placement and taxonomic assignment of *C. challengeri*, we aligned the amino acids of all 13 protein-encoding genes available from Amphipoda mitogenomes (111 circular and 87 linear) using MUSCLE 3.8.425 (Edgar 2004), and masked the alignment to remove any position containing >30% gaps. A maximum likelihood phylogenetic tree was constructed from this alignment using the PROTGAMMAJTT model in RAxML 8.2.12, with 300 bootstrap replicates (Stamatakis 2014). The resulting phylogenetic tree was plotted using Iroki (Moore et al. 2020). References for mitogenomes used in the phylogenetic tree are provided in Supplemental Table 1.

## Results

**Figure 2.**
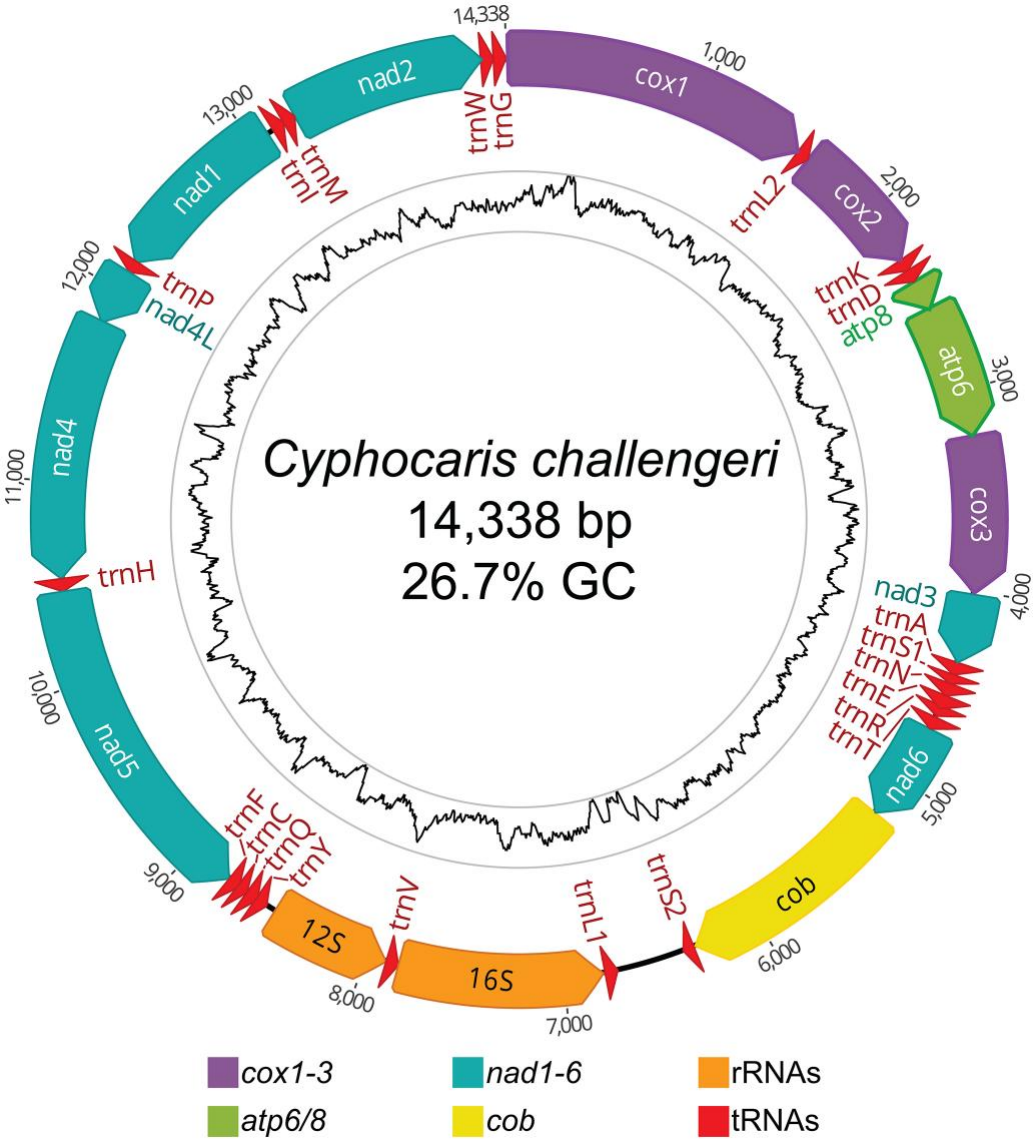
Circular mitogenome map of *Cyphocaris challengeri*. All annotated genes are indicated with their direction. Inner tract shows GC content with an 80 bp sliding window; high (51% GC) and low (5% GC) values are bounded by the gray rings.

The assembled circular mitogenome of *C. challengeri* (accession OQ064431) is 14,338 bp long with an average GC content of 26.7% (35.5% A, 37.9% T, 18.0% C, 8.7% G). The mitogenome contains 13 protein-encoding genes, 2 ribosomal RNAs, and 22 tRNAs (Figure 2). Four different start codons were found: ATG (*n* = 5), ATT (*n* = 4), TTG (*n* = 2), and ATA (*n* = 2). The control region is likely located between the *trnL1* and *trnS2* genes, in an AT-rich (85.8%) 339 bp non-coding region with poly-T motifs, though experimental evidence is required to confirm. The mitochondrial genome was reordered from the original assembly to begin with the start codon for cytochrome oxidase I (COI). In addition to the mitogenome, we recovered two haplotypes of the complete nuclear rRNA-ITS region (accessions OQ093270 and OQ093271), with haplotype B containing a 5 bp and 16 bp insertion in the ITS2 region compared to haplotype A (see notes on Genbank record).

**Figure 3.**
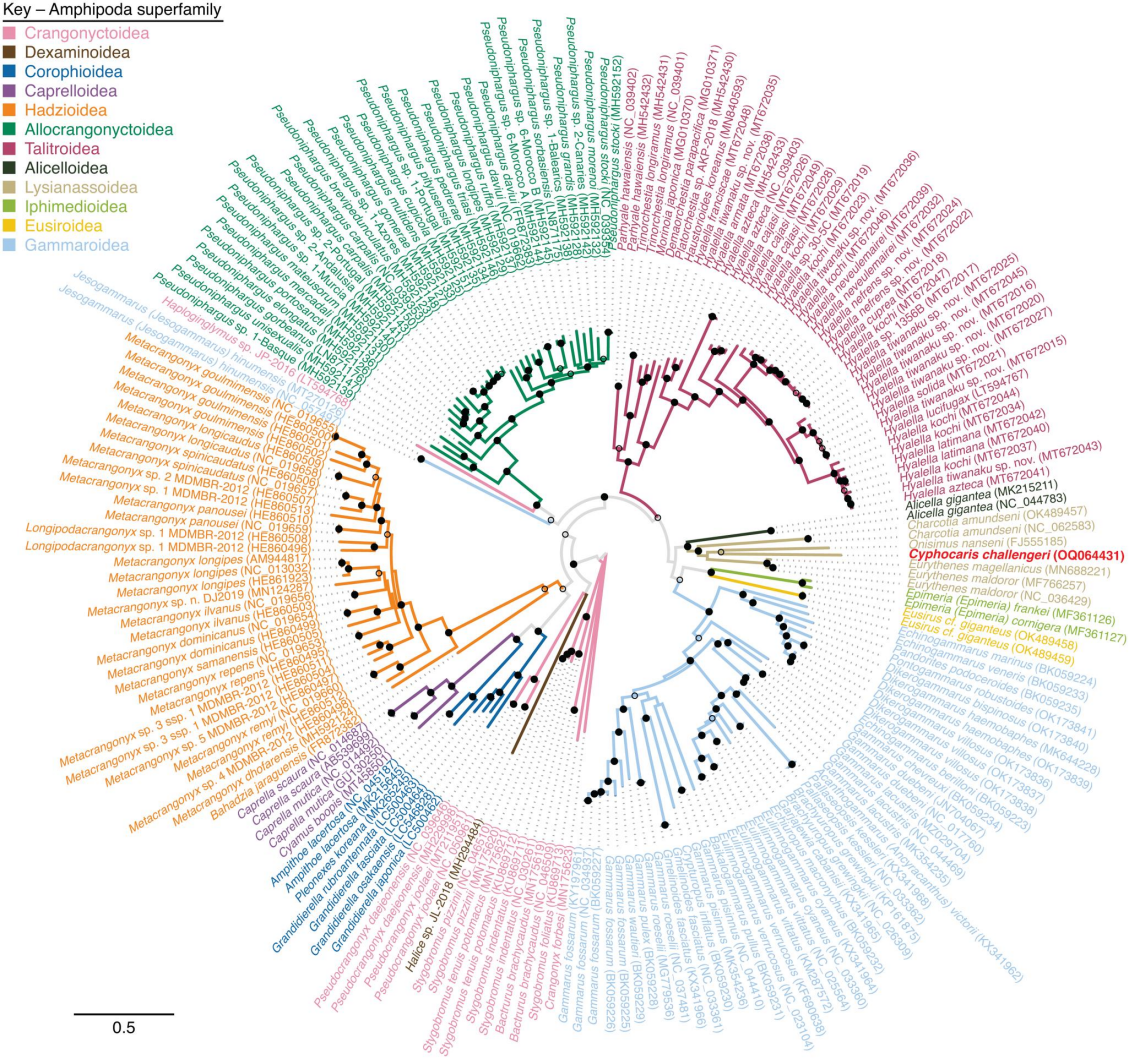
Maximum likelihood phylogenetic tree of a concatenated alignment of all thirteen protein encoding genes available from Amphipoda mitogenomes. *Cyphocaris challengeri* label is highlighted in red. Branches are colored by superfamily. Bootstrap values are indicated at nodes with a solid dot (≥75% support), open dot (≥50%, <75% support), or no dot (<50% support).

Phylogenetic analysis puts *C. challengeri* as a sister taxon to *Onisimus nanseni* (Sars 1900), with the next closest relatives in the genus *Eurythenes* (Smith 1882) (Figure 3). As expected, the closest relatives of *C. challengeri* all reside within the superfamily Lysianassoidea (Dana 1849), forming a monophyletic clade. A similarly constructed tree based on nucleotide sequences and adding the rRNA genes showed a similar topology, with the exception of the two *Jesogammarus (Jesogammarus) hinumensis* (Morino 1993) sequences, which fall basal to the Lysianassoidea with low bootstrap support (data not shown). The protein-encoding and rRNA gene orientation and order for *C. challengeri* most closely resemble other amphipods from the parvorder Lysianassidira, particularly *Eurythenes magellanicus* (Milne Edwards 1848), *Eurythenes maldoror* (D'Udekem d'Acoz and Havermans 2015), and *Alicella gigantea* (Chevreux 1899). However, its closest relative, *O nanseni*, does not share this mitogenome order.

## Discussion and conclusion

The complete mitochondrial genome and nuclear rRNA-ITS region of *C. challengeri* presented in this study is the first complete genomic reference data for the Cyphocarididae family. Currently, only partial-length COI sequences are registered in NCBI (*Cyphocaris bouvieri* (Chevreux 1916), *Cyphocaris tunicola* (Lowry and Stoddart 1997), and non-speciated BOLD voucher specimens). Thus, this work will enhance our ability to detect this species and its close relatives in environmental DNA (eDNA) observatories, improving ecosystem monitoring and modeling (Mathieu et al. 2020).

## Supplementary Material

Supplemental Material

Supplemental Material

## Data Availability

The complete mitochondrial genome of *Cyphocaris challengeri* is available on NCBI under accession number OQ064431. The complete rRNA-ITS region of two haplotypes (A/B) is available under accession number OQ093270 and OQ093271, respectively. The raw sequencing data from the *C. challengeri* genome is available through NCBI’s SRA, under BioProject, SRA, and BioSample accession numbers PRJNA913968, SRR22972441, and SAMN32316678, respectively.
