# Supplementary figures and images for: The complete mitochondrial genome of Cyphocaris challengeri (Amphipoda: Cyphocarididae)
Source: Mitochondrial DNA B Resour. 2023 Oct 25;8(10):1128–31. doi: 10.1080/23802359.2023.2270206 (PMC10621271; doi:10.1080/23802359.2023.2270206)

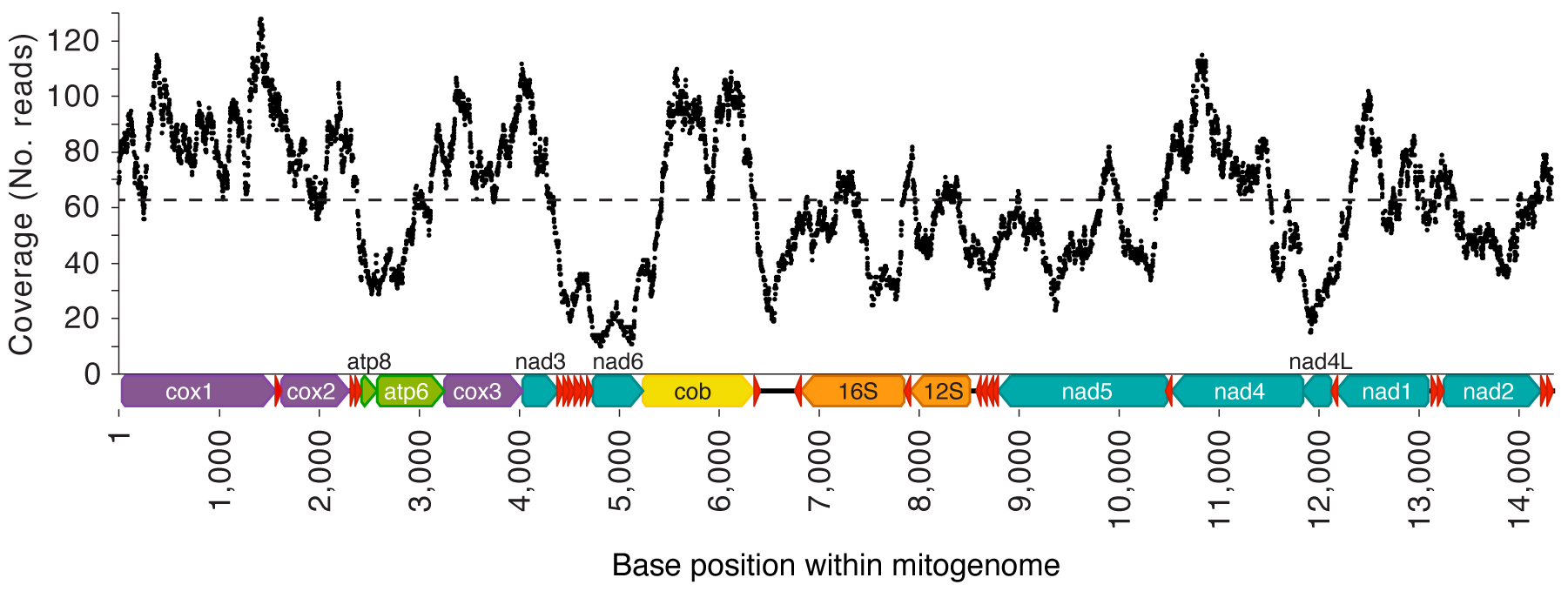

Supplement: Supplemental Material [file TMDN_A_2270206_SM1036.tif]
